# Resistance to tumour challenge after tumour laser thermotherapy is associated with a cellular immune response

**DOI:** 10.1038/sj.bjc.6602718

**Published:** 2005-08-09

**Authors:** K Ivarsson, L Myllymäki, K Jansner, U Stenram, K-G Tranberg

**Affiliations:** 1 Department of Surgery, Lund University, SE-22185 Lund, Sweden; 2 Department of Pathology, Lund University, SE-22185 Lund, Sweden

**Keywords:** interstitial laser thermotherapy, CD4 and CD8 lymphocytes, ED1 and ED2 macrophages, immune response

## Abstract

Previous studies in our laboratory have shown that interstitial laser thermotherapy (ILT) of an experimental liver tumour is superior to surgical excision, at least partly due to a laser-induced immunological effect. The aim of the present study was to investigate the time–response relationship of the ILT-induced immunisation and the cellular response of macrophages and lymphocytes. A dimethylhydrazine-induced adenocarcinoma was transplanted into the liver of syngeneic rats. Rats with tumour were treated 6–8 days later (tumour size 0.25–0.40 cm^3^) with ILT of tumour or resection of the tumour-bearing lobe. Two groups of rats without tumour were treated with resection of a normal liver lobe or ILT of normal liver. A challenging tumour was implanted into the liver of each rat 2, 5 or 10 weeks after primary treatment. Rats were killed 6, 12 and 48 days (or earlier due to their condition) after challenge (*n*=8 in all groups). Immunohistochemical techniques were used to determine lymphocytes (CD8, CD4) and macrophages (ED1, ED2) in rats having had treatment of a primary tumour. Interstitial laser thermotherapy of the first tumour was followed by eradication of challenging tumour and absence of tumour spread. This contrasted with rapid growth and spread of challenging tumour in the other groups. In the challenging vital tumour tissue and in the interface between the tumour and surroundings, the number of ED1 macrophages and CD8 lymphocytes was higher in rats having been treated with the ILT of tumour than in those having undergone resection of the tumour-bearing lobe. The number of ED2 macrophages and CD4 lymphocytes was low and did not vary between these two groups. Interstitial laser thermotherapy elicited an immune response that eradicated a challenging tumour and was associated with increased numbers of tumour-infiltrating macrophages and CD8 lymphocytes.

In the future, curative treatment of solid tumours is likely to consist of local treatment (surgical excision or some form of local tissue destruction) coupled with different types of adjuvant therapy. The reason why local excision alone may not be sufficient for cure is the risk for occult disseminated disease and, also, the risk for local recurrence. Surgery is known to induce immunosuppression and the release of growth factors that may stimulate growth of residual tumour (discussed in Möller *et al*, 1998). The reason why systemic therapy like chemotherapy or immunotherapy is likely to be insufficient as the sole treatment is their relative inefficacy for solid tumours and the fact that clinical tumour burdens are large. As for immunotherapy, possible success appears to depend on combination with procedures that give efficient tumour killing and antigen exposure. Heating of tumour cells with interstitial laser thermotherapy (ILT) is interesting in this respect, since it can accomplish total local tumour destruction by tumour necrosis at the same time as it induces an immunological response against the tumour (Möller *et al*, 1998; Tranberg *et al*, 2002).

Using a model of adenocarcinoma implanted into rat liver, we reported that ILT gave results that were superior to those of surgical resection of the tumour-bearing liver lobe (Möller *et al*, 1998). The efficacy of local tumour removal was similar, but the ILT method was associated with less extrahepatic spread. Evidence for a laser-induced immunological effect was then obtained by the following study. Using inoculation of tumour cell suspensions into the lateral and median lobes of the liver simultaneously and treating the lateral lobe tumour only, we found that laser thermotherapy reduced take and growth of the untreated tumour in the median lobe. This twin tumour study thus provided direct evidence that the advantageous effect of ILT can at least partly be explained by the induction of an immunological response (Tranberg *et al*, 2002).

The aim of the present study was to examine the time–response relationship of the ILT-induced immunisation and to elucidate the possible mechanisms by studying tissue densities of macrophages and CD4 and CD8 lymphocytes.

## MATERIAL AND METHODS

### Animals and tumour

The experiments were performed with male rats from Møllegaard A/S (Ejby, Denmark), but complementary studies were performed with Wistar FU from our own breeding. Rats weighed 224–292 g at the time of tumour implantation. They were housed individually and had free access to standard food pellets (Ewos R3; Lactamin AB, Södertälje, Sweden) and tap water *ad libitum*. The tumour was a dimethylhydrazine-induced, weakly immunogenic adenocarcinoma of the rat colon obtained from the Wallenberg Research Laboratory, Lund (Steele and Sjögren, 1974). The tumour was propagated by weekly intraperitoneal passages in inbred Wistar rats from our own breeding. Generations 140–154 were used in this study.

Tumour implantation was performed with vital and macroscopically homogenous tumour. At midline laparatomy and using an operating microscope (Olympus OMK 1, Olympus Optical, Tokyo, Japan), a 3-mm-long and 2-mm-deep incision was made on the anterior surface of the liver. A piece of Spongostan (Ferrosan A/S, Søborg, Denmark) was applied to the incision area for 5 min to control bleeding. A piece of the tumour (1.0 mm in diameter) was then implanted into the parenchyma, followed by application of Spongostan for another 2 min. The first tumour (the tumour to be treated) was implanted into the left lateral lobe of the liver, whereas the second (challenging) tumour was implanted into the median lobe of the liver. Tumour preparations were made immediately before transplantation and were kept on ice cubes until implanted.

Rats were anaesthetised with an intraperitoneal injection of 5% chloral hydrate (0.5 mg per 100 g body weight) for tumour implantation. Ether was used for all other procedures that required anaesthesia, including treatments, challenges and euthanasia. All treatments were performed using a midline abdominal incision. Treatment procedures were followed by intramuscular injection of buprenorphine (Temgesic, Meda AB, Göteborg, Sweden), 0.002 mg per 100 g body weight, for analgesia.

Animal procedures were performed according to the recommendations of the Swedish Board of Animal Research and were approved by the Committee of Animal Ethics at Lund University.

### Experimental protocol

We used 288 rats which were randomly allocated to four groups. Two groups of rats with tumour (I–II) were treated with ILT of tumour (I) or resection of the tumour-bearing lobe (II). Two groups of rats without tumour (III–IV) were treated with resection of a normal liver lobe (III) or ILT of normal liver (IV). Treatment of rats with tumour (I–II) was performed 6–8 days after implantation, at which time tumour had a size of 140±25 (mean±s.e.m.) mm^3^ and did not vary between the treatment groups (*P*>0.05). A challenging tumour was implanted into the liver of each rat 2, 5 or 10 weeks after primary treatment (groups I–IV). Rats were planned to be killed 6, 12 and 48 days after challenge (*n*=8 in all groups). However, rats were euthanised as soon as they showed signs of inactivity or distress. At that time, there was usually palpable intraabdominal tumours.

After killing, the liver, lungs, peritoneum and all tissues containing suspect tumour growth were harvested for microscopic examination. Immunohistochemical examinations were carried out after challenge in rats having had treatment of a primary tumour (groups I–II).

### Interstitial laser thermotherapy (ILT)

**Figure 1:**
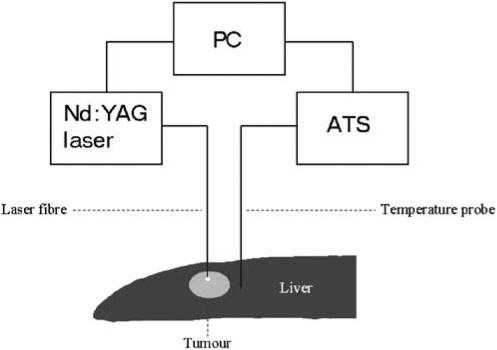
Schematic illustration of feedback temperature system for ILT. ATS, automatic thermometry system; PC, personal computer.

Thermotherapy was carried out with a system consisting of an Nd:YAG laser and a temperature feedback control unit interfaced with the laser, as described in detail elsewhere (Möller *et al*, 1996, 1997, 1998) (Figure 1). The laser beam was delivered through a 600-*μ*m flexible bare fibre at a laser output power of 2 W. The bare fibre was placed at right angles into the centre of the tumour, at a depth measuring one-third of the thickness of the liver, including the tumour. A feedback thermistor probe (Microtherm AB, Lund, Sweden) was placed perpendicularly into the liver parenchyma at a distance of 3 mm from the tumour margin and at a depth of 2 mm from the liver surface. The distance from the tip of the bare fibre to the thermistor probe averaged 6.4±0.1 (mean±s.e.m.) mm.

Interstitial laser thermotherapy was performed at a steady-state target temperature of 46°C for 30 min at the thermistor probe, that is, 3 mm from the tumour margin. The treatment characteristics were based on previous studies defining the temperature and duration of treatment needed for complete tumour necrosis, as judged 6 days after treatment (Möller *et al*, 1996, 1997, 1998). Interstitial laser thermotherapy of normal liver was performed in the same way, using the distance between the laser fibre and thermistor probe found in rats with tumours.

### Resectional procedures

Resection of the tumour-bearing, or normal, left lateral lobe was performed after division of the falciform ligament and the triangular plicae on the left side. A ligature of 4-0 Suturamid (Johnson and Johnson AB, Sweden) was tied around the most central part of the venous and arterial pedicle to the left lateral liver lobe. The liver was transected with a pair of scissors 1–2 mm distal to the ligature and 10.3±0.4 (mean±s.e.m., range 7.0–15.0) mm proximal to the tumour, as measured macroscopically. A soft gauze was carefully placed under the left lateral liver lobe in order to collect possible bleeding from the resection line.

### Histopathology

Liver tissues were fixed in 4% paraformaldehyde in 0.1 M sodium phosphate buffer (PB) as a rule for 24 h. The livers with tumours were delivered uncut to the pathologist, who examined the specimens while unaware of the treatment. A section was cut through what was considered to be the largest diameter of the lesion(s), sometimes supplemented with one or two additional sections. The specimens were then rinsed, dehydrated and embedded in paraffin. After sectioning, the slides were processed for immunohistochemistry or stained with haematoxylin/eosin.

The sizes of total tumour and necrotic tumour were measured under the microscope with a micrometer, because it was impossible to distinguish necrosis, inflammatory changes and tumour with the naked eye, especially in rats that had been treated with laser thermotherapy.

### Immunohistochemistry

The sections were deparaffinised with xylene and brought to 96% alcohol and washed in 0.1 M sodium phosphate-buffered saline (PBS) with 0.25% Triton X-100, pH 7.2. Immunohistochemical reaction for tissue macrophages was performed with ED1 and ED2 mouse anti-rat antibodies (diluted 1 : 200) (Serotec Ltd, Oxford, UK), using a secondary rabbit anti-mouse antibody (Dijkstra *et al*, 1985; Damoiseaux *et al* 1994). Reaction for CD4 and CD8 was performed with mouse anti-rat antibodies (diluted 1 : 200) (Nordic Biosite AB, Täby, Sweden), using a secondary rabbit anti-mouse antibody (Whiteland *et al*, 1995). Visualisation was performed with diaminobenzidine (DAB) (Saveen Biotech AB, Malmö, Sweden) (brown colour). Counterstaining was made with haematoxylin *ad modum* Mayer. Negative controls (buffer instead of primary antibody) were performed to each reaction.

Labelled cells were counted per visual field with an × 40 objective and an × 10 ocular. As a rule, only whole fields were counted on peripheral vital tumour and granulation tissue. It was tried to count six fields for each type of tissue, but that was not always possible. For each rat, ED1, ED2, CD4 and CD8 slides were counted one after the other to ensure that the same areas were counted with all antibodies. The order of examination of slides varied. Necroses and large vessels were avoided. The mean per visual field was used in the statistical analysis.

### Calculations

Lesion size (*V*) was estimated according to the formula *V*=*a* × *b*^2^/2, where *a* is the largest width and *b* is the maximum diameter perpendicular to the width of the tumour (Carlsson *et al*, 1983). Before treatment, these measurements, including measurement of the liver thickness, were performed during laparotomy using a vernier caliper.

The significance of differences between groups was assessed with the Mann–Whitney test. Values are means±s.e.m. Probabilities of less than 0.05 were accepted as significant.

## RESULTS

The tumour is a poorly differentiated carcinoma growing expansively in the liver without or with very little tissue reaction from the host. Tumours that were treated with resection had irregular necroses in the centre and were proliferating in the periphery with a rather smooth boundary to the surrounding liver tissue without inflammatory reaction, except for an occasional small area of granulation tissue. The microscopic changes seen a week after laser treatment have been described in detail in previous communications (Möller *et al*, 1996, 1997, 1998). Briefly, the treated tumour contains cells with faintly stained nuclei and eosinophilic cytoplasm, as a rule surrounded by a layer of necrotic liver tissue. In the peripheral parts of the necroses, there are polymorphonuclear leucocytes and macrophages and a few lymphocytes.

The growth of challenging liver tumours in groups I–II is summarised in Table 1. The challenging tumour after ILT of tumour (group I) was followed by eradication of reimplanted tumour at 48 days and absence of tumour spread. After 48 days, there was as a rule only a fibrous scar, included in Table 2 under ‘tumour capsule’, sometimes no remnant at all. This contrasted with the rapid growth and spread of tumour following implantation of tumour in the other groups (II–IV), leading to euthanasia of all animals within 30 days. In two of the ILT animals vital tumour cells were found at 48 days at immunohistochemistry, but not in haematoxylin–eosin sections. None of the rats that had undergone ILT of the first tumour showed evidence of ascites or extrahepatic spread (group I). All other rats had both extensive intraperitoneal tumour growth and ascites, with no obvious differences between rats that had undergone resection of the tumour-bearing left lateral liver lobe, resection of the normal left lateral liver lobe and ILT of normal liver (groups II–IV).

With the exception of the ILT animals, the tumours that were implanted into treated rats had the same appearance as the tumours that were resected before reimplantation. In rats treated with ILT, there appeared to be relatively large numbers of polymorphonuclear leucocytes in the peripheral parts of necrotic tumour.

The number of ED1 macrophages in tumour capsule and viable tumour was significantly larger in the ILT (group I) than in the resection groups (II) at 12 days after challenge (*P*<0.05 in both instances). The difference between the ILT and resection groups was more obvious for CD8 lymphocytes: numbers were larger in the ILT group both at 6 and 12 days after challenge, and both in the tumour capsule and in viable tumour (*P*<0.05 in all instances). ED2 and CD4 cells were always few, and there were no differences between the ILT and resection groups (*P*>0.05) (Table 2). There was often a concentration of ED1 cells in the tumour periphery.

**Figure 2:**
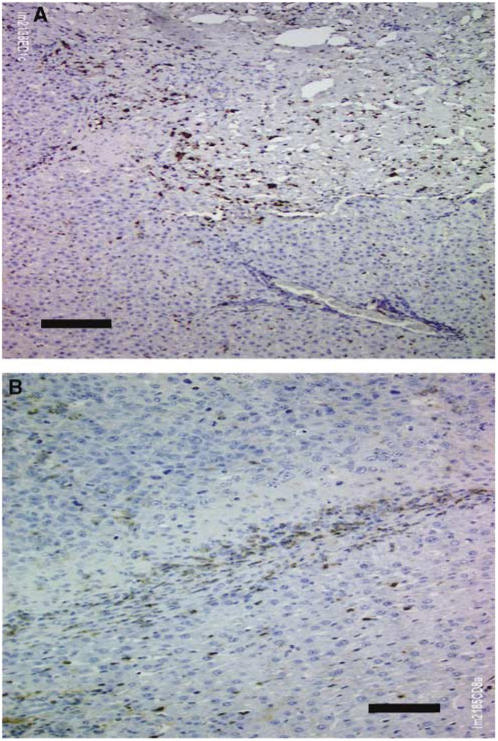
Immunohistochemical findings 12 days after rechallenge following ILT (group I). (**A**) Fibrotic remnants of tumour in the upper half, liver tissue in the lower half. Brown colour indicates ED1 macrophages. Black bar: 50 *μ*m. (**B**) Vital tumour from another animal, in the upper third of the photo, liver tissue in the lower half. Brown colour indicates CD8 lymphocytes. Black bar: 50 *μ*m.

As a rule, ED1- and ED2-positive cells had the appearance of macrophages in vital tumour (Figure 2). In the granulation tissue, myofibroblast-like cells as well as macrophages were positive for both ED1 and ED2 antibodies. Generally, more macrophages than myofibroblast-like cells were positive. The number of macrophages in granulation tissue should be regarded with some caution, but it appears that ED1-positive cells should represent mainly macrophages in vital tumour tissue.

## DISCUSSION

Local tumour therapy with hyperthermia focuses on two different aspects: (a) atraumatic reduction or ablation of tumour (Tranberg *et al*, 1996, 2002; Möller *et al*, 1998; Vogl *et al*, 1998; Isbert *et al*, 2002), and (b) induction of tumour immunity, alone or together with cytokine therapy (Möller *et al*, 1998; Tranberg *et al*, 2002; Dranoff, 2004). This study confirmed that laser thermotherapy can elicit a favourable immunological response at the same time as it eradicates the primary tumour, and showed that this response remains strong at least 10 weeks after treatment. It also showed that laser treatment was followed by an immune cellular response of tumour-infiltrating macrophages and CD8 lymphocytes.

CD8 lymphocytes and ED1 macrophages invaded the challenging tumour. This agrees with findings in studies in which antitumour immunity has been induced and where these two cell types have been found to be of importance (Caruso *et al*, 1993; Barba *et al*, 1994; Consalvo *et al*, 1995; Yamamoto *et al*, 1997; Xiang *et al*, 1998; Kuriyama *et al*, 1999a, 1999b). Antitumour immunity is a complex phenomenon. There is growing evidence that dendritic cells are important for induction of immunity (Todryk *et al*, 1999; Basu *et al*, 2000; Sauter *et al*, 2000). Other studies have suggested that CD4 cells (Caruso *et al*, 1993; Kuriyama *et al*, 1999a, 1999b), natural killer cells (Pierrefite-Carle *et al*, 1999) and antibodies (Chen *et al*, 1999) also may play important roles. Futhermore, it appears that recognition of tumour antigens by T lymphocytes is not enough for efficient tumour cell killing and for elimination of the effect of tumour-induced immunosuppression (Horiguchi *et al*, 1999). Efficient immune therapy seems to require a local inflammatory reaction of a certain type coupled with the secretion of a mixture of appropriate cytokines (Ganss *et al*, 2002; Dranoff, 2004). The local presence of cytokines like IL-12, IL-18, INF-*γ* or TNF-α helps to generate cytolytic effector cells.

Though we and the cited authors have used a syngeneic tumour model, it can be argued that the tumour was raised a long time ago, in our system 30 years ago. A genetic drift may have occurred, in the tumour and/or the rats, and transplantation antigens may have been responsible, at least in part, for the outcome. However, in our previous twin tumour study, the same effect was obtained in rats that were inoculated with a pure tumour cell suspension at two different sites in the liver (Tranberg *et al*, 2002), a model that carries a reduced risk of transplantation antigen involvement. In addition, we performed experiments also in another breed of the same syngeneic strain, and were pleased to obtain the same results (data not shown). It would be of considerable interest to examine two quite different tumours in the same animal strain with similar or identical transplantation antigens.

Some of the elongated myofibroblast-like cells in the tumour capsule-granulation tissue stained positive with the ED1, and occasionally the ED2, macrophage and CD8 antibodies. Fibroblasts and endothelial cells have been described occasionally to be positive for ED1 (Damoiseaux *et al*, 1994). In the normal liver surrounding the tumour, we found plenty of elongated sinusoidal cells positive with ED1. The most plausible explanation is that they are Kupffer cells, but we cannot exclude that some might be endothelial cells. Perhaps these elongated, ED1-positive cells might be incorporated into the capsule.

A vaccination effect has been suggested to be important for the effect of the two chemotherapy models cytosine deaminase/5-fluorocytosine (Consalvo *et al*, 1995; Kuriyama *et al*, 1999b; Morris, 1999; Pierrefite-Carle *et al*, 1999) and herpes simplex virus thymidine kinase/ganciclovir (Barba *et al*, 1994; Yamamoto *et al*, 1997; Kuriyama *et al*, 1999a), and for the effect of photodynamic therapy (PDT) (Chen *et al*, 1997; Chen *et al*, 1999). These modalities induce tumour cell necrosis, and an inflammatory microenvironment, which is critical for eliciting an efficient immune response (Melcher *et al*, 1998; Todryk *et al*, 1999; Sauter *et al*, 2000). Cell death following irradiation, ‘conventional’ chemotherapy and conventional hyperthermia (⩽42.5°C) is mainly apoptotic (Cummings *et al*, 1997), which should give a less immunogenic response.

There is strong evidence that heat shock proteins (HSPs) are involved in mediating the immunological response against tumours. Heat shock protein–tumour peptide complexes are taken up by antigen-processing cells like macrophages/dendritic cells, where the tumour peptides are processed and presented to T cells, eliciting an immune response (Basu *et al*, 2000; Sauter *et al*, 2000; Srivastava, 2002). In our tumour model, ILT causes a shift of HSP70 from the cytoplasm to nucleus in tumour cells, an increase in tumour-infiltrating macrophages and an increase of HSP70 in ED1-positive macrophages (Ivarsson *et al*, 2003). It has been shown that necrotic, but not apoptotic, cell death can cause the release of HSPs (Basu *et al*, 2000; Sauter *et al*, 2000; Srivastava, 2002).

The reason why we use the low temperature of 46°C 3 mm from the tumour margin (during 30 min) is that we want to obtain cell death that develops into necrosis within a time range of hours to a few days (Wheatley *et al*, 1989). It is important that there is no coagulation of protein at this temperature level, which means that undestroyed tumour antigens can be exposed to the immune system. Furthermore, at this temperature, tumour blood flow is not abolished (Sturesson *et al*, 2005). Nevertheless, the ILT method produces complete necrosis of treated tumour (Möller *et al*, 1997, 1998; Tranberg *et al*, 2002).

In conclusion, laser thermotherapy appears to be a promising method for curative treatment of solid tumours. Hyperthermia may play an important role in immunotherapeutic approaches in the future.

## Figures and Tables

**Table 1:** Size of challenging tumour after treatment (*n*=8 in each group)

		**Vital tumour at killing (mm^3^), days after implantation of new tumour**		
**Group**	**Implantation post-treatment, weeks**	**6 days**	**12 days**	**48 days^a^**
I. Interstitial laser thermotherapy (ILT) of tumour	2	34±15	201±36	1.0±1.0^b^
	5	25±5.6	150±66	0
	10	6.6±3.4	15±14	0
				
II. Resection of tumour-bearing liver lobe	2	280±11	204±25	2493±405
	5	336±16	1836±249	1321±250
	10	231±44	1787±341	1779±339
				
III. Resection of normal liver lobe	2	351±33	426±81	2372±396
	5	282±25	1741±311	2319±381
	10	354±16	2272±319	2843±365
				
IV: Interstitial laser thermotherapy (ILT) of normal liver	2	270±15	841±196	3117±385
	5	366±48	2381±351	3333±201
	10	267±36	1472±86	3473±372

Values are means±s.e.m.

a Rats having been treated with ILT of tumour survived for 48 days after challenge with new tumour. All other rats had to be euthanised within 10–30 days with extensive tumour burden, intraperitoneal spread and ascites; these rats were included in the 48-days column.

b Two tumours.

**Table 2:** Density of ED1 and ED2 macrophages and CD4 and CD8 lymphocytes in implanted tumour in treated rats (*n*=8 in each group)

		**ED1-positive macrophages^a^**		**ED2-positive macrophages^a^**		**CD4 lymphocytes^a^**		**CD8 lymphocytes^a^**	
**Group**	**Implantation post-treatment, weeks/killing, days after challenge**	**Tumour capsule**	**Viable tumour**	**Tumor capsule**	**Viable tumour**	**Tumour capsule**	**Viable tumour**	**Tumour capsule**	**Viable tumour**
I. Interstitial laser thermotherapy (ILT)									
	2w/6d	76±25	74±24	0.2±0.4	0.8±1.0	0.8±0.8	0±0	14±8.3*	15±7.6*
	5w/6d	58±14	54±19	0±0	0.6±0.5	0±0	0±0	27±16*	20±22*
	10w/6d	65±29	79±13	0.5±0.5	0.5±0.5	—	0.3±0.5	17±23*	9.8±6.1*
									
	2w/12d	76±35*	64±29*	0.4±0.5	—	0.5±0.9	0±0	29±24*	11±15*
	5w/12d	73±19*	73±31*	0±0	0.6±1.0	0.1±0.3	0±0	20±12*	18±15*
	10w/12d	62±15	65±22	1.1±1.1	0.8±1.2	1.4±1.3	0.2±0.4	12±13	26±10
									
	2w/48d^b^	19±23	—	0.3±0.6	0.5±0.7	—	—	3.3±4.2	0.5±0.7
	5w/48d	46±26	—	0±0	—	0±0	—	1.1±1.2	—
	10w/48d	37±12	—	1.0±1.4	—	1.0±1.4	—	2.5±2.1	—
									
II. Resection of tumour-bearing liver lobe									
	2w/6d	77±20	73±18	0.3±0.8	0.1±0.4	0.3±0.5	0.7±0.8	13±14	10±5.2
	5w/6d	63±13	62±12	1.2±1.6	1.5±0.8	0±0	0±0	17±16	2.3±3.4
	10w/6d	91±20	89±29	0.25±0.5	1.0±1.4	0.5±0.6	0.6±0.5	11±5.9	14±7.8
									
	2w/12d	64±25	54±29	0.9±1.6	0.4±0.5	0±0	0±0	14±12	12±8.6
	5w/12d	58±14	49±13	0.3±0.5	0.2±0.4	0.2±0.4	0.2±0.4	17±8.4	11±10
	10w/12d	—	—	—	—	—	—	—	—
									
	2w/48d^c^	—	—	—	—	—	—	—	—
	5w/48d^c^	25±21	0	1.0±1.7	1.0±1.4	1.0±1.4	1.0±1.4	1.3±1.1	0±0
	10w/48d^c^	—	—	—	—	—	—	—	—

w=weeks; d=days.

a Number of cells per visual field.

b Two rats with signs of vital tumour in the liver.

c Animals had to be euthanised 10–30 days after reimplantation and only two tumours were analysed. Values are means±s.e.m.

* *P*<0.05 for comparisons between ILT and resection of tumour-bearing liver lobe. For comparison between treatments, values were pooled for each post-treatment challenge day, since there were no differences within each day for different weeks after treatment.
